# Risk Factors for Low Receptive Vocabulary Abilities in the Preschool and Early School Years in the Longitudinal Study of Australian Children

**DOI:** 10.1371/journal.pone.0101476

**Published:** 2014-07-02

**Authors:** Daniel Christensen, Stephen R. Zubrick, David Lawrence, Francis Mitrou, Catherine L. Taylor

**Affiliations:** Telethon Kids Institute, The University of Western Australia, Perth, Western Australia, Australia; University Children's Hospital Tuebingen, Germany

## Abstract

Receptive vocabulary development is a component of the human language system that emerges in the first year of life and is characterised by onward expansion throughout life. Beginning in infancy, children's receptive vocabulary knowledge builds the foundation for oral language and reading skills. The foundations for success at school are built early, hence the public health policy focus on reducing developmental inequalities before children start formal school. The underlying assumption is that children's development is stable, and therefore predictable, over time. This study investigated this assumption in relation to children's receptive vocabulary ability. We investigated the extent to which low receptive vocabulary ability at 4 years was associated with low receptive vocabulary ability at 8 years, and the predictive utility of a multivariate model that included child, maternal and family risk factors measured at 4 years. The study sample comprised 3,847 children from the first nationally representative Longitudinal Study of Australian Children (LSAC). Multivariate logistic regression was used to investigate risks for low receptive vocabulary ability from 4–8 years and sensitivity-specificity analysis was used to examine the predictive utility of the multivariate model. In the multivariate model, substantial risk factors for receptive vocabulary delay from 4–8 years, in order of descending magnitude, were low receptive vocabulary ability at 4 years, low maternal education, and low school readiness. Moderate risk factors, in order of descending magnitude, were low maternal parenting consistency, socio-economic area disadvantage, low temperamental persistence, and NESB status. The following risk factors were not significant: One or more siblings, low family income, not reading to the child, high maternal work hours, and Aboriginal or Torres Strait Islander ethnicity. The results of the sensitivity-specificity analysis showed that a well-fitted multivariate model featuring risks of substantive magnitude does not do particularly well in predicting low receptive vocabulary ability from 4–8 years.

## Introduction

The initiation and expansion of language from childhood onwards is a universal and remarkable developmental accomplishment [1]. Receptive vocabulary is a central marker of language development as well as general ability and literacy. From onset in infancy, it develops rapidly in the preschool and school years, from around 200 words in the second year [2], to 20,000 words at 8 years [3]. Because vocabulary is also characterized by its continued growth throughout life, it has featured as a prominent and longstanding measure frequently used in omnibus scales of ability and intelligence [4], [5]. To the extent that measures of vocabulary predict general ability and literacy they are also associated with onward academic competency and success [6].

Longitudinal studies have shown a consistent pattern of early emergence of disparities in language acquisition that persist over time [7] and have far reaching consequences for children's success at school and opportunities beyond school [8], [9], [10], [11], [12]. Because of the crucial role of oral language as a developmental means for literacy, education and employment, language acquisition is one of the major pathways that is seen to support human capability formation [13].

The rapid growth in receptive vocabulary in the preschool and school years invites a careful consideration of the underlying mechanisms that drive this growth. Necessarily, investigations of predictors of variation in the onset and growth of language abilities require large numbers of children and collection of data on a broad range of candidate predictors. The trade-off is that measures of language ability are generally narrow because direct behavioural assessment of children's language abilities is expensive. Such large-scale studies are rare in the field of language acquisition and largely designed to investigate a number of key developmental outcomes, not just language development. Therefore, in population level studies, measurement is typically restricted to one component of language [1], [12], [14] or one composite measure of general language ability [9], [15]. This stands in contrast to smaller, ‘purposive’ studies which often measure specific aspects of language in great detail but do not necessarily capture growth or change within an ecological context.

A recent study of receptive vocabulary growth from 4–8 years showed substantial variation in receptive vocabulary ability at 4, 6 and 8 years and that predictor significance and strength also varied over time [1]. The study produced some counterintuitive effects: For example, factors associated with low receptive vocabulary ability at 4 years of age were maternal Non-English Speaking Background (NESB), low school readiness, child not read to at home, four or more siblings, low family income, low birthweight, low maternal education, maternal mental health distress, low maternal parenting consistency, and high child temperament reactivity. Yet, none of these factors were associated with an onward lower rate of growth from 4–8 years. Instead, the following factors were associated with a *higher* rate of receptive vocabulary growth: Maternal NESB, low school readiness and maternal mental health distress, although these higher rates of growth did not completely close the receptive vocabulary ability gap for children with and without these risks. The only risk factor associated with a lower onward rate of growth was socio-economic area disadvantage, which was actually not a risk for low receptive vocabulary ability at the intercept (i.e., 4 years) [1].

In a study that used data from the 1970 British Cohort Study, low receptive vocabulary at 5 years was a significant risk factor for low functional literacy in adulthood [12]. Five-year-olds with receptive vocabulary ability 1 SD below the mean (11.5% of the sample), were more than 3 times as likely to have low functional literacy in adulthood compared to 5-year-olds who met normative expectations for receptive vocabulary ability. The odds were substantially higher for children with receptive vocabulary ability 2 SD below the mean (3.9% of the sample). These children were almost 7 times as likely to have low functional literacy in adulthood, compared to children who met normative expectations for receptive vocabulary ability at 5 years. However, low receptive vocabulary ability at 5 years did not foretell low functional literacy attainment for most children. Sixty-eight percent of the 5-year-olds with low receptive vocabulary ability, defined as 1 SD below the mean, attained onward functional literacy. Once again, and somewhat counterintuitively, the percentage of children who attained functional literacy was higher for children who performed 2 SD below the mean than children who performed 1 SD below the mean. Eighty percent of children with low receptive vocabulary ability 2 SD below the mean attained functional literacy, compared to 68% of children with low receptive vocabulary ability 1 SD below the mean [12]. In a study that used data from the Children of the NLSY79 [14], race disparities in receptive vocabulary ability evident at 5 years persisted through to 13 years.

What remains unknown is the extent to which low language ability persists or improves beyond toddlerhood, and the extent to which other factors in the child's wider developmental environment are associated with this in the preschool and early school years. This study had two aims. First, to investigate the extent to which low receptive vocabulary at 4 years was associated with onward low receptive vocabulary at 8 years. Second, to estimate the predictive utility of a multivariate model that included child, maternal and family risk factors measured at 4 years toward the prediction of low language ability at 8 years. This study offers a rare assessment of a comprehensive set of risk factors for the persistence of low receptive vocabulary in kindergarten- and school-age children in a representative population level sample.

## Methods

### Ethics Statement

The Longitudinal Study of Australian Children (LSAC) is conducted in a partnership between the Australian Government Department of Social Services (DSS), the Australian Institute of Family Studies (AIFS) and the Australian Bureau of Statistics (ABS). The study has ethics approval from the Australian Institute of Family Studies Ethics Committee. The Ethics Committee is registered with the Australian Health Ethics Committee, a subcommittee of the National Health and Medical Research Council (NHMRC). Caregivers gave written informed consent to the survey. As the study children were all minors at the time these data were collected, written informed consent was obtained from the caregiver on behalf of each of the study children. The signed consent forms are retained by the field agency (ABS).

### Access and Use of LSAC Data

Confidentialised LSAC data are publicly available. Researchers can apply to the Australian Government Department of Social Services for permission to access and use Longitudinal Study of Australian Children (LSAC) data (DSS website. Available: http://www.dss.gov.au/our-responsibilities/families-and-children/programs-services/growing-up-in-australia-the-longitudinal-study-of-australian-children-lsac. Accessed 2014 Feb 7Aug 2).

### Study Design

The study sample comprised 3,847 children who participated in the Longitudinal Study of Australian Children (LSAC). The LSAC is a national longitudinal study that commenced in 2004. Data collection for the study is led by a consortium of expert Australian researchers [16]. Guided by a bioecological model of child development [17], data are collected on child, parental, family, community and school characteristics that influence children's development at different ages (i.e., a developmental pathways approach) [18], [19]. The measurement framework is comparable to indicator frameworks used internationally [20]. Indicator frameworks group variables that influence child development into key domains. For example, time, income, human capital, psychological capital and social capital [21]. This has produced a comprehensive (and expanding) set of independent variables that researchers can select from, to model in relation to specific developmental outcomes, which in this study, is language development.

The study uses a cross-sequential design of biennial face-to-face visits with the family and study child. In this study we used data from the child cohort collected at 4 and 8 years. The child cohort comprised 4,983 children at 4 years and 4,331 children at 8 years. Response rates to the outcome measure, the Adapted Peabody Picture Vocabulary Test-III (PPVT), and the candidate predictors varied; 4,406 children completed the PPVT at 4 years, 4,273 completed the PPVT at 8 years; and 3,847 children completed the PPVT at 4 and 8 years.

The LSAC sampling frame was extracted from the Medicare Australia enrolment database, which was validated to ensure coverage of Australian children within the target age-range. The initial study sample was designed to be representative of Australian children within the selected age cohort, proportional to the regional distribution of children in the Australian population. An initial sample size of 5,000 was chosen as to ensure there would still be a sufficient sample for detailed analysis after attrition over the number of years of the longitudinal study.

The study entailed a two-stage clustered design, first selecting postcodes then children within postcodes. Stratification was used to ensure proportional geographic representation for states/territories and capital city statistical division/rest of state areas. Cluster sampling was utilised because it provides a cost effective way to conduct face-to-face interviews, as well as an opportunity to collect and analyse community-level effects. Postcodes were selected with probability proportional to size selection where possible, and with equal probability for small population postcodes. Children were selected from 311 postcodes [22], [23].

Analyses show that the initial sample was broadly representative of the general Australian population when compared with 2001 Census data, but slightly under-representative of families who were single-parent, non-English speaking and living in rental properties. Attrition somewhat increased these biases. For example, the overall attrition rate between ages 4 and 8 was 13%, but children with mothers classified as Non-English speaking background decreased from 15.7% at age 4 to 13.8% at age 8, an attrition rate of 23%. The proportion of mothers who had a year 11 or less education decreased from 39.2% at age 4 to 36.5% at age 8, an attrition rate of 19% [24].

### Measures

The selection of measures used in this paper is guided by the LSAC ethos, empirical and/or theoretical associations with language development, and our previous work in the area.

The LSAC was funded as part of the (then) Department of Family and Community Services Stronger Families and Communities Strategy, which aimed to establish partnerships to strengthen families and communities and develop and deliver solutions at a local level [25]. The survey was designed with an explicit aim of use by a range of Commonwealth and State and Territory departments, and the research community. The variables selected for inclusion in the LSAC were based upon known risk factors from established longitudinal studies and a consideration of those variables which could meaningfully impact policy interventions [25].

Our variable selection in this paper is guided by our previous work in this area [1]. The study conceptual model [17], [26] posited multiple domains of influence on child development. Among these domains are characteristics related to the child, the mother, and the family home environment. Variables in this predictor set met one of the following two criteria: (1) Evidence of an independent association with English language abilities in a representative population level sample of preschool and school age children; or (2) conceptual relevance to language abilities, in the absence of empirical evidence. Many of the measures are benchmarked against Australian census collections while others are referenced to large scale Australian and international child development studies.

Our previous work, using a multi-level modelling approach to receptive vocabulary growth, revealed some counter-intuitive findings [1]. In the multivariate model, risks for receptive vocabulary delay at 4 years, in order of magnitude, were: Maternal Non- English Speaking Background (NESB), low school readiness, child not read to at home, four or more siblings, low family income, low birthweight, low maternal education, maternal mental health distress, low maternal parenting consistency, and high child temperament reactivity. None of these risks were associated with a lower rate of growth from 4–8 years. Instead, maternal NESB, low school readiness and maternal mental health distress were associated with a higher rate of growth, although not sufficient to close the receptive vocabulary gap for children with and without these risks at 8 years. Socio-economic area disadvantage, was not a risk for low receptive vocabulary ability at 4 years but was the only risk associated with a lower rate of growth in receptive vocabulary ability. These variables were detailed extensively in that paper. The majority of these are single-item measures.

#### Response variable

Our response variable is a measure of one the major component systems of language development – the semantic system. Children were assessed with the Adapted Peabody Picture Vocabulary Test-III (PPVT), a test of receptive vocabulary designed for the LSAC study [27]. The Adapted PPVT-III is a shortened version of the PPVT–III [28]. The Adapted PPVT-III was developed and validated specifically for Australian children at 4, 6 and 8 years, and the technical properties are described in detail by Rothman [27], [29]. The Pearson product-moment correlation between the full PPVT-III and the Adapted PPVT-III was 0.93 for all children. After administration to the LSAC children, a one-parameter (Rasch) item response model was fitted to the data, which consisted of correct and incorrect responses, this gave the Adapted PPVT-III a person-separation reliability of 0.76 [27], [29].

The Adapted PPVT-III was administered directly to each child during the home interview. For each word presented, the child was shown a card containing four pictures and was asked to point to the picture corresponding to the word (e.g., “Show me wrapping”). Scaled scores for the Adapted PPVT-III were used in all analyses. Table 1 contains the median ages and age ranges in months for the study children along with the mean Adapted PPVT-III Rasch scaled score with SDs and associated ranges.

**Table 1:** Children's ages and PPVT scores by longitudinal wave and sample size.

Wave (N)	Child's age in months			PPVT scores		
	Median	Range	Mean		SD	Range
1 (4983)	57	51–67	65		6	28–85
2 (4464)	82	75–94	74		5	46–92
3 (4331)	105	95–119	78		5	45–106

#### Age standardizing

**Figure 1:**
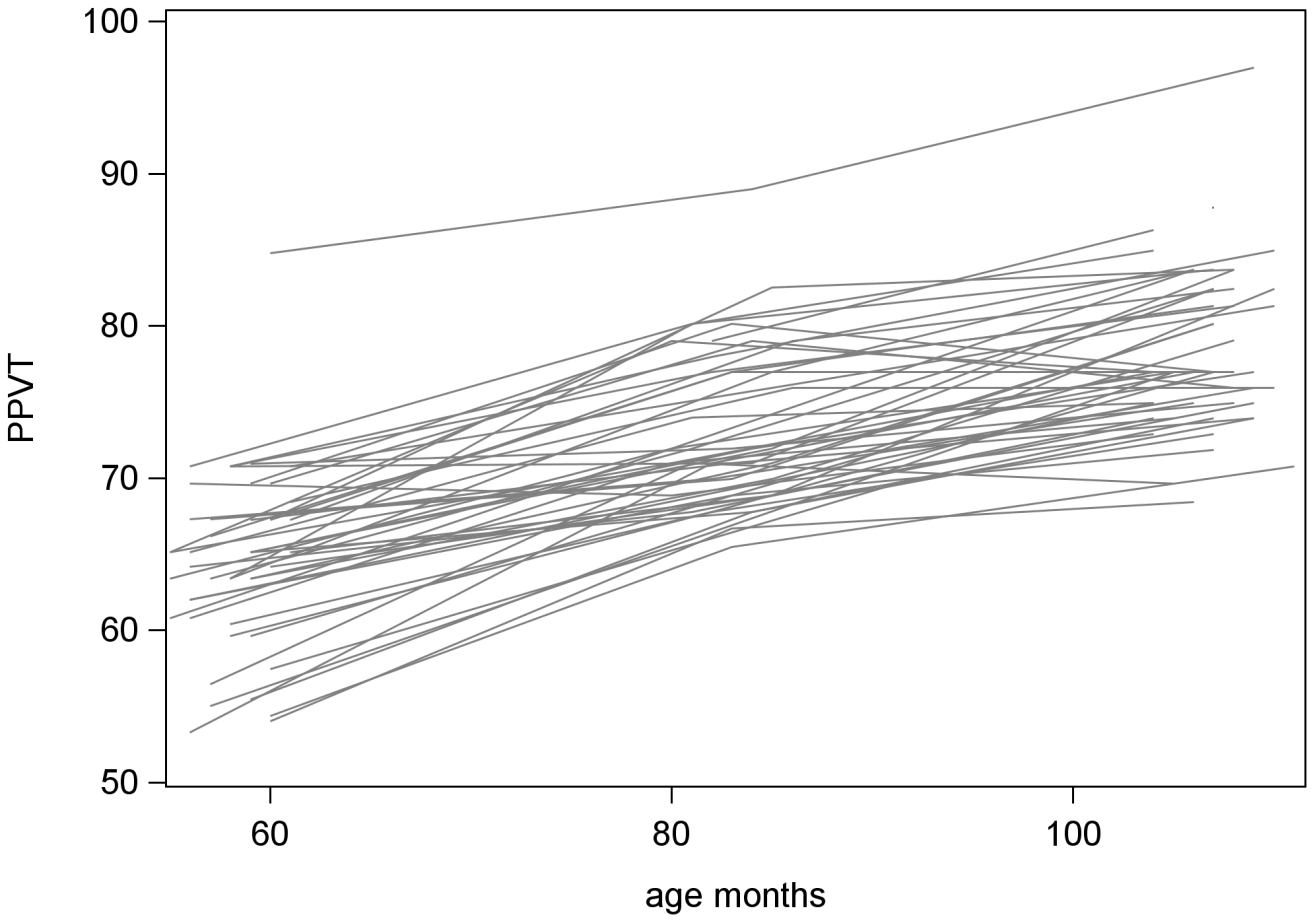
Empirical growth plots (n = 50).

In this study we wanted to examine whether children's receptive vocabulary, relative to their peers, at age 4 (51–67 months) is predictive of their receptive vocabulary, relative to their peers, at age 8 (95–119 months). Our previous work [1] established the relationship between child age and receptive vocabulary (PPVT), as illustrated in Figure 1. A linear regression model estimated a slope of 0.35 at age 4; that is, for every month older the study children get, we expect an increase of 0.35 in their PPVT scores. Therefore, we decided to adjust for child age, by comparing children's receptive vocabulary to their peers closest to them in age.

To do this we created age-groups of approximately equal size within the 4-year and 8-year age-cohorts (see Table 2). We then created PPVT z-scores where children were ranked relative to their peers within each of these age groups. Once this was completed we re-assembled the PPVT z-score rankings for each of the age 4-year and age 8-year cohorts. Thus, a child at 51 months of age who is -2 standard deviations on the PPVT measure compared to their age peers can be compared with a child of 59 months who is also -2 standard deviations on the PPVT measure. A similar method was used by Sanson et al [30] in establishing outcome measures for the LSAC.

**Table 2:** Age-groups within the 4-year and 8-year age-groups.

4-year age-group		8-year age group	
age-group (months)	n	age-group (months)	n
51–54	940	95–102	670
55–56	1292	103–104	921
57–58	1363	105–106	1160
59–60	943	107–108	846
61–67	445	109–119	734
*Total*	*4983*		*4331*

Finally, as we were interested in the risks of low receptive vocabulary, we defined the children in each of the age 4 and age 8 cohorts as having low receptive vocabulary where they fell at or below the lowest 15^th^ percentile in PPVT performance. This enabled us to ask whether, relative to their peers, having low receptive vocabulary at 4 years is predictive of their receptive vocabulary at 8 years.

#### Candidate predictors

A total of 29 candidate predictor variables were used. These were grouped into child, maternal, and family and home environment characteristics. All predictor variables were measured when the child was 4 years of age. Predictors were modelled as risk variables with the lowest level of risk as the reference category (see Table 3). For each variable, the percentage of the sample in each of the reference categories, along with the percentage of the sample in each of the other categories are shown in Table 3. For example, in examining birthweight we used children of ‘normal’ birthweight as our reference group. For school readiness, we used children in the quintile with the highest *Who Am I?* scores as our reference group. The analytic sample for each candidate predictor varied somewhat, depending on item completeness.

**Table 3:** Child, maternal and family variables.

*Child variables*	%	*Maternal variables*	%
**Sex**		**Mother's age at birth**	
Male	50.9	Teen	2.9
Female (ref.)	49.1	40+	3.2
		20–39 (ref.)	93.9
**Ethnicity**			
Study Child ATSI^a^	3.8	**Mother alcohol problem**	
Study Child non-ATSI (ref.)	96.2	Yes	12.1
		No (ref.)	87.9
**Birth weight**			
Low birth weight	6.5	**Mother smoker**	
Normal birth weight (ref.)	93.5	Yes	23.1
		No (ref.)	76.9
**Ear infections**			
Yes	7.9	**Mother K6 symptomatic**	
No (ref.)	92.1	Yes	16.2
		No (ref.)	83.8
**Who Am I?**			
Quintile 1 (lowest)	19.3	**Maternal education**	
Quintile 2	22.4	Year 12	32.3
Quintile 3	16.4	Year 11 or less	39.2
Quintile 4	20.5	University (ref.)	28.5
Quintile 5 (highest) (ref.)	21.4		
		**Maternal work hours**	
**Persistence**		Zero hours^b^	43.3
Quintile 1 (lowest)	18.9	Full time: 38 hours+	13.3
Quintile 2	25.8	Part time: 1–37 hours (ref.)	43.4
Quintile 3	22.7		
Quintile 4	17.4	**Maternal consistency**	
Quintile 5 (highest) (ref.)	15.2	Quintile 1 (least consistent)	21.7
		Quintile 2	16.1
**Reactivity**		Quintile 3	21.9
Quintile 1 (most reactive)	21.9	Quintile 4	20.4
Quintile 2	17.7	Quintile 5 (ref.)	19.9
Quintile 3	20.4		
Quintile 4	21.0	**Maternal inductive reasoning**	
Quintile 5 (ref.)	19.0	Quartile 1 (lowest reasoning)	16.8
		Quartile 2	33.7
**Sociability**		Quartile 3	22.7
Quintile 1 (lowest)	18.4	Quartile 4 (ref.)	26.8
Quintile 2	24.4		
Quintile 3	21.1	**Maternal warmth**	
Quintile 4	19.2	Quintile 1 (lowest warmth)	21.4
Quintile 5 (ref.)^a^	16.9	Quintile 2	24.4
		Quintile 3	11.9
		Quintile 4	23.2
		Quintile 5 (ref.)	19.1
		**Maternal hostility**	
		Quintile 1 (greatest hostility)	21.6
		Quintile 2	27.8
		Quintile 3	16.4
		Quintile 4	17.7
		Quintile 5 (ref.)	16.5

*Family variables*	%		%
**Mother Non-English speaking background**		**Health care card**	
Yes	15.7	Yes	22.0
No (ref.)	84.3	No (ref.)	78.0
**Family structure**		**Financial hardship**	
Single mother family	14.0	Yes	30.6
Other (ref.)	86.0	No (ref.)	69.4
**Number of siblings**		**SEIFA disadvantage index**	
One	48.4	Quintile 1 (lowest SEIFA)	21.9
Two	26.7	Quintile 2	20.8
Three	9.3	Quintile 3	19.4
Four or more	4.1	Quintile 4	18.6
Zero (ref.)	11.5	Quintile 5 (ref.)	19.3
**Family income**		**Reads to child**	
Under $600	17.5	Not at all	3.6
$600–$999	23.9	1–2 days/ week	19.5
$1000–$1499	25.0	3–5 days/ week	29.7
$1500–$1999	17.0	Daily (ref.)	47.2
$2000 or more (ref.)	16.6		
		**Playgroup**	
		No	67.8
		Yes (ref.)	32.2
		**Hours a week in care**	
		9–20	65.5
		21–30	20.0
		31+	8.2
		8 or less hours (ref.)	6.3

a Aboriginal and Torres Strait Islander;

b Includes not in labour force.

The child characteristics in our models were: Gender, ethnicity, birth weight, ear infections, school readiness and temperament. There were equal proportions of girls and boys in the sample. A small proportion of children (n = 121; 2.8%) were of Aboriginal and/or Torres Strait Islander decent and were coded to distinguish them from those who were not. Primary carers were asked to report their child's birth weight which was subsequently coded into those children who were born with low birth weight (<2500 grams; 6.3%) and those who weighed more than this (> = 2500 grams). A single item indicator of ongoing ear infections at 4 years was included.

In addition to the Adapted PPVT-III, each study child was directly assessed at 4 years using the *Who Am I?* [31]. This is a measure of school readiness and comprises 11 items in which children write their names, copy shapes and write words and numbers. It has been extensively calibrated for use in the LSAC and has well demonstrated item characteristics, high internal reliability (0.89), and excellent distributional properties [29]. Rothman [29] reported a correlation between the Adapted PPVT-III and the *Who Am I?* of 0.31 for the LSAC children at age 4. In this report, study children have been grouped into quintiles of performance based on the total *Who Am I?* score with high quintiles representing higher levels of performance.

Child temperament was measured at 4 years with the Short Temperament Scale for Children (STSC) [32]. The STSC measures three dimensions of temperament: persistence, reactivity and sociability. Each temperament dimension was assessed through parent report using four items, rating the frequency of the behaviours on a 6-point Likert scale of occurrence from “almost never” to “almost always”. Where data were missing for any of the items making up a dimension of temperament respondents were coded as missing for that variable. Four composites were constructed based on the respective items and each was then divided into quintiles with higher quintiles representing the positive aspects of each dimension.

The maternal characteristics in our models were: Age at the birth of the child, problematic alcohol use, smoking, mental health distress, education, hours of paid employment and parenting. The biological mother's age at the birth of the child was grouped into categories representing teen birth (< age 20 years), 20–39 years and 40 or more years at birth with the vast majority of mothers (94.5%) of study children in the age range 20–39 years.

Information on current tobacco and alcohol was gathered from the mothers. We defined problematic alcohol use where women reported their daily alcohol consumption to exceed 2 standard drinks and/or where they reported frequent binge drinking of 5 or more alcoholic drinks at least 2–3 times per month with 12.0% being so classified. Study children's mothers were asked about tobacco use and also categorised as either current smokers (21.8%) or not current smokers.

In this study, we used the Kessler-6 (K-6) scale to measure maternal non-specific psychological distress. Women with scores of 8 or more were classified as having symptomatic psychological distress. This threshold is consistent with other studies [33], [34] using the K-6. The scale has robust characteristics as an indicator of mental health with recent Australian findings [35]. Fifteen per cent of mothers reported symptomatic psychological distress.

In Australia, at the time of this study, 10 years of education was compulsorily mandated. Maternal education in years was grouped into three levels according to those who had completed 11 years (36.4%), 12 years (32.9%), and those who had completed more than 12 years (i.e. University education) (30.7%).

Mothers were variously employed at the time when the children were first measured. We used total hours of paid maternal employment to distinguish mothers who were not in paid employment (0 hours), from those in part time paid employment (1–37 hours; Mean  = 17.8) and in full time paid employment (> = 38 hours; Mean  = 44.9). Similar proportions of women were either not in paid employment (41.0%) or working part time (45.5%) with the remainder (13.5%) working full time.

The parenting characteristics of both parents were measured in a self-complete form, using four measures of parenting warmth, hostility, consistency and inductive reasoning developed for the LSAC [36]. We use the mother's responses in this report. Responses to each item were on a 5-point Likert scale, ranging from “almost never” to “always/almost always”. Items for each measure were summed to create a composite score with higher levels representing more positive parenting characteristics. Item and scale properties for the LSAC parenting measures have been extensively documented [37]. Ordinal scale reliabilities [38] were 0.72 for maternal hostility, 0.82 for consistency and 0.93 and 0.94 for warmth and inductive reasoning respectively.

The characteristics of the family home environment in our models were: NESB, family structure, number of siblings, income, health care card, financial hardship, socioeconomic disadvantage, reading to the study child, playgroup and child care. As the focus of this study is explicitly on English language development and because language development is known to vary where more than one language is spoken in the home, we used the mother's Non-English Speaking Background (NESB) as a general indicator for language other than English spoken in the household at 4 years. About 14% of mothers were predominately non-English speaking at the time of the interview.

With respect to family composition, two variables were selected as candidate predictors of vocabulary development: Family structure (sole parent vs. other) and number of siblings (0, 1, 2, 3, 4+). About 11.5% of the study children were living in single mother families at 4 years. The majority had one or two siblings (49.5% and 27.4% respectively) or were singletons (10.9%) at age 4. The study questionnaire did not permit the establishment of birth order.

Families were asked to report their total weekly family income from all sources. Responses were partitioned into relatively equal quintiles: those families earning under $600, $600–$999, $1000–$1499, $1500–$1999, and $2000 or more per week. In Australia where income falls below a defined threshold and/or certain hardship criteria are met families also qualify for a health care card. About 19.7% of LSAC families had a health care card and this is used as an indicator of financial need in the LSAC families. Additionally, an indicator of family hardship was also derived where families reported, due to shortage of money over the last 12 months that: they had not been able to pay gas, electricity or telephone bills on time; they had not been able to pay the mortgage or rent on time; adults or children had gone without meals; they had been unable to heat or cool their home; they had pawned or sold something; or sought assistance from a welfare or community organisation. About thirty per cent of families reported at least one of these occurrences in the previous 12 months at 4 years.

An area measure of socioeconomic disadvantage was also estimated for each participating family. The family home was coded with Socio-Economic Indicators for Area (SEIFA) disadvantage, indexed in quintiles – lower quintiles represent greater levels of disadvantage. The neighbourhood SEIFA disadvantage index summarizes information from the Australian Census of Population and Housing as this relates to economic and social disadvantage in small areas, such as low income, low educational attainment and high unemployment [39]. These data were linked at the Statistical Local Area (SLA) level or, where this was not available, the child's postcode.

Several indicators of the child's learning environment were gathered. The frequency with which the primary caregiver read to the study child was assessed via face-to-face interview. A total of 129 (3.0%) parents reported not reading to the child at all, 802 (18.8%) reported reading 1 or 2 days a week, 1,255 (29.4%) reported reading to the child 3–5 days a week, and 2085 (48.8%) reported reading to the child daily. Mothers were asked if their child had attended a playgroup in the 12 months prior to the 4-year interview with about one third indicating this to be the case. Finally, hours per week in child care or early education were identified by asking the primary carer “how many hours a week on average does the child go to school, kindergarten, pre-school, and/or day-care?”. A total of 258 (6.3%) attended 8 or less hours a week, 2,705 (65.8%) attended 9–20 hours a week, 818 (19.9%) attended 21–30 hours a week, and 332 (8.1%) attended 31or more hours a week.

Table 4 shows the child, maternal and family home environment variables measured at age 4 and the percentage of the sample, at different levels of risk in the low PPVT group versus the middle-high PPVT group at 8 years. For example, of the 778 children who scored in the lowest quintile for school readiness (*Who Am I?*) at age 4, 25.7% were in the low PPVT group at age 8. In contrast, of the 935 children in the most favourable quintile for school readiness (*Who am I?*) at age 4, only 8.3% were in the low PPVT group at age 8. Of the 511 children who scored in the low PPVT group at age 4, 37.6% were in the low PPVT group at age 8, while of the 612 children tested in the middle-high PPVT group at age 4 only 2.6% were in the low PPVT group at age 8. The latter observation is important as it illustrates that while low PPVT at age 4 indicates an increased risk of low PPVT at age 8, the majority of children with low PPVT at age 4 moved into the middle-high group at age 8.

**Table 4:** The child, maternal and family characteristics of children in the low PPVT and middle-high PPVT groups at 8 years.

Predictor variables	Low PPVT at 8 years (n = 638)	Middle-high PPVT at 8 years (n = 3635)
**Child Characteristics at age 4**		
**PPVT group**		
Low (bottom 15%) (n = 511)	192 (37.6%)	319 (62.4%)
Middle (65%) (n = 2723)	314 (11.5%)	2409 (88.5%)
High (top 15%) (n = 612) (ref.)	16 (2.6%)	596 (97.4%)
**Study Child Sex**		
Male (n = 2178)	328 (15.1%)	1850 (84.9%)
Female (n = 2095) (ref.)	310 (14.8%)	1785 (85.2%)
**Ethnicity**		
Study Child ATSI^a^ (n = 121)	38 (31.4%)	83 (68.6%)
Study Child Non-ATSI (n = 3943) (ref.)	599 (14.4%)	3551 (85.6%)
**Birth weight**		
Low birth weight (n = 265)	49 (18.5%)	216 (81.5%)
Normal birth weight (n = 3956) (ref.)	573 (14.5%)	3383 (85.5%)
**Study Child Ear Infections**		
Yes (n = 330)	55 (16.7%)	275 (83.3%)
No (n = 3943) (ref.)	583 (14.8%)	3360 (85.2%)
**Who Am I? (quintiles)**		
1 (lowest) (n = 778)	200 (25.7%)	578 (74.3%)
2 (n = 949)	149 (15.7%)	800 (84.3%)
3 (n = 690)	89 (12.9%)	601 (87.1%)
4 (n = 867)	102 (11.8%)	765 (88.2%)
5 (highest) (n = 935) (ref.)	78 (8.3%)	857 (91.7%)
**Persistence (quintiles)**		
1 (lowest) (n = 668)	147 (22%)	521 (78%)
2 (n = 968)	134 (13.8%)	834 (86.2%)
3 (n = 845)	102 (12.1%)	743 (87.9%)
4 (n = 647)	84 (13%)	563 (87%)
5 (highest) (n = 570) (ref.)	54 (9.5%)	516 (90.5%)
**Reactivity (quintiles)**		
1 (most reactive) (n = 783)	139 (17.8%)	644 (82.2%)
2 (n = 639)	93 (14.6%)	546 (85.4%)
3 (n = 765)	102 (13.3%)	663 (86.7%)
4 (n = 786)	97 (12.3%)	689 (87.7%)
5 (n = 703) (ref.)	84 (11.9%)	619 (88.1%)
**Sociability (quintiles)**		
1 (lowest) (n = 687)	122 (17.8%)	565 (82.2%)
2 (n = 897)	129 (14.4%)	768 (85.6%)
3 (n = 764)	115 (15.1%)	649 (84.9%)
4 (n = 721)	89 (12.3%)	632 (87.7%)
5 (n = 631) (ref.)	64 (10.1%)	567 (89.9%)
**Maternal Characteristics at age 4**		
**Mother's age at birth**		
Teen (n = 97)	23 (23.7%)	74 (76.3%)
40 and over (n = 134)	13 (9.7%)	121 (90.3%)
20–39 (n = 3990) (ref.)	586 (14.7%)	3404 (85.3%)
**Mother alcohol problem**		
Yes (n = 433)	72 (16.6%)	361 (83.4%)
No (n = 3171) (ref.)	420 (13.2%)	2751 (86.8%)
**Mother smoker**		
Yes (n = 804)	154 (19.2%)	650 (80.8%)
No (n = 2890) (ref.)	364 (12.6%)	2526 (87.4%)
**Mother K6 symptomatic**		
Yes (n = 559)	102 (18.2%)	457 (81.8%)
No (n = 3112) (ref.)	411 (13.2%)	2701 (86.8%)
**Maternal education**		
Year 12 (n = 1543)	332 (21.5%)	1211 (78.5%)
Year 11 or less (n = 1397)	204 (14.6%)	1193 (85.4%)
University (n = 1302) (ref.)	94 (7.2%)	1208 (92.8%)
**Maternal work hours**		
zero hours^b^ (n = 1754)	333 (19%)	1421 (81%)
full-time: 38 hours + (n = 575)	81 (14.1%)	494 (85.9%)
part time: 1–37 hours (n = 1944) (ref.)	224 (11.5%)	1720 (88.5%)
**Maternal consistency (quintiles)**		
1 (least consistent) (n = 844)	192 (22.7%)	652 (77.3%)
2 (n = 673)	107 (15.9%)	566 (84.1%)
3 (n = 923)	109 (11.8%)	814 (88.2%)
4 (n = 890)	124 (13.9%)	766 (86.1%)
5 (n = 887) (ref.)	90 (10.1%)	797 (89.9%)
**Maternal inductive reasoning (quartiles)**		
1 (lowest reasoning) (n = 690)	131 (19%)	559 (81%)
2 (n = 1441)	192 (13.3%)	1249 (86.7%)
3 (n = 984)	138 (14%)	846 (86%)
4 (n = 1103) (ref.)	161 (14.6%)	942 (85.4%)
**Maternal warmth (quintiles)**		
1 (lowest warmth) (n = 915)	133 (14.5%)	782 (85.5%)
2 (n = 1044)	140 (13.4%)	904 (86.6%)
3 (n = 500)	82 (16.4%)	418 (83.6%)
4 (n = 985)	151 (15.3%)	834 (84.7%)
5 (n = 774) (ref.)	116 (15%)	658 (85%)
**Maternal hostility (quintiles)**		
1 (greatest hostility) (n = 903)	161 (17.8%)	742 (82.2%)
2 (n = 1161)	170 (14.6%)	991 (85.4%)
3 (n = 702)	99 (14.1%)	603 (85.9%)
4 (n = 754)	108 (14.3%)	646 (85.7%)
5 (n = 697) (ref.)	84 (12.1%)	613 (87.9%)
**Family home environment characteristics at age 4**		
**Mother Non-English speaking background**		
yes (n = 589)	138 (23.4%)	451 (76.6%)
no (n = 3657) (ref.)	493 (13.5%)	3164 (86.5%)
**Family structure**		
Single mother family (n = 491)	102 (20.8%)	389 (79.2%)
Other (n = 3782) (ref.)	536 (14.2%)	3246 (85.8%)
**Number of siblings**		
only child (n = 2113) (ref.)	276 (13.1%)	1837 (86.9%)
1 sibling (n = 1172)	180 (15.4%)	992 (84.6%)
2 siblings (n = 376)	75 (19.9%)	301 (80.1%)
3 siblings (n = 148)	52 (35.1%)	96 (64.9%)
4 or more siblings (n = 464)	55 (11.9%)	409 (88.1%)
**Family income**		
under $600/week (n = 601)	126 (21%)	475 (79%)
$600–999 (n = 932)	175 (18.8%)	757 (81.2%)
$1,000–$1,499 (n = 1060)	144 (13.6%)	916 (86.4%)
$1,500–$1,999 (n = 727)	95 (13.1%)	632 (86.9%)
$2,000 or more/week (n = 704) (ref.)	50 (7.1%)	654 (92.9%)
**Health care card**		
yes (n = 840)	183 (21.8%)	657 (78.2%)
no (n = 3433) (ref.)	455 (13.3%)	2978 (86.7%)
**Financial hardship**		
yes (n = 1207)	222 (18.4%)	985 (81.6%)
no (n = 3062) (ref.)	415 (13.6%)	2647 (86.4%)
**SEIFA disadvantage index (quintiles)**		
1 (lowest SEIFA) (n = 897)	211 (23.5%)	686 (76.5%)
2 (n = 893)	149 (16.7%)	744 (83.3%)
3 (n = 830)	113 (13.6%)	717 (86.4%)
4 (n = 811)	91 (11.2%)	720 (88.8%)
5 (n = 842) (ref.)	74 (8.8%)	768 (91.2%)
**Reads to child**		
no reading (n = 129)	47 (36.4%)	82 (63.6%)
1–2 days/ week (n = 802)	168 (20.9%)	634 (79.1%)
3–5 days/ week (n = 1255)	207 (16.5%)	1048 (83.5%)
daily (n = 2085) (ref.)	216 (10.4%)	1869 (89.6%)
**Playgroup**		
yes (n = 2509)	370 (14.7%)	2139 (85.3%)
no (n = 1215) (ref.)	155 (12.8%)	1060 (87.2%)
**Hours a week in care**		
9–20 (n = 2705)	381 (14.1%)	2324 (85.9%)
21–30 (n = 818)	136 (16.6%)	682 (83.4%)
31+ (n = 332)	57 (17.2%)	275 (82.8%)
0–8 (n = 258) (ref.)	36 (14%)	222 (86%)

a Study child Aboriginal and/or Torres Strait Islander status;

b Includes not in labour force.

### Data Analysis

The data were analysed using logistic regression in SAS 9.3 [40]. The surveylogistic procedure was used to account for the complex survey design of the LSAC. Logistic regression was used to estimate the odds of children being in the low group of PPVT (lowest 15% in each age-group) at wave 3 of the LSAC (age 7–9 years).

Our analysis proceeded in four steps. First, we used logistic regression to estimate the association between each of our candidate predictors and PPVT group (i.e., low vs. middle-high) at 8 years. Second, we grouped candidate predictors by their estimated effect size. Third, we established a multivariate logistic regression model. Finally, we undertook a sensitivity-specificity analysis to examine the predictive utility of our multivariate model.

To establish the effect sizes for the various predictors we used the odds ratios as estimated by logistic regression. For logistic regression, the odds ratio itself represents the effect size of interest [41]. Although some schemes exist denoting a correspondence between odds ratios and a substantive effect size (e.g. [41], [42]), the judgement of what denotes a substantive effect size rests with the researcher, and must be considered within the context of the field of study [43]. For this paper we established an odds ratio of 2 or greater as our cut-off for a moderate effect size.

## Results

### Bivariate Analysis

Table 5 contains the odds ratios for low receptive vocabulary at 8 years (bottom 15%), based on the candidate predictors of low performance at 4 years.

**Table 5:** Bivariate associations between child, maternal and family characteristics and low receptive vocabulary ability at 8 years.

Predictor Variables	OR	P-value
Child Variables		
**PPVT**		
Low (bottom 15%)	5.32 (4.36–6.49	
Middle-high (Remaining 85%) (ref.)	1.00	<.0001
**Gender**		
Male	1.03 (0.88–1.21)	
Female (ref.)	1.00	0.70
**Ethnicity**		
Study Child ATSI^a^	2.67 (1.65–4.30)	
Study Child non-ATSI (ref.)	1.00	<.0001
**Birth weight**		
Low Birth weight	1.17 (0.85–1.62)	
Normal Birth weight (ref.)	1.00	0.325
**Ear Infections**		
Yes	1.13 (0.83–1.55)	0.4392
No (ref.)	1.00	
**Who Am I?**		
quintile 1 (lowest)	3.79 (2.93–4.91)	
quintile 2	2 (1.54–2.59)	
quintile 3	1.62 (1.2–2.2)	
quintile 4	1.38 (1.03–1.83)	
quintile 5 (ref.)	1.00	<.0001
**Persistence**		
quintile 1 (lowest)	2.75 (1.97–3.84)	
quintile 2	1.54 (1.1–2.15)	
quintile 3	1.32 (0.96–1.82)	
quintile 4	1.59 (1.11–2.28)	
quintile 5 (ref.)	1.00	<.0001
**Reactivity**		
quintile 1 (most reactive)	1.75 (1.32–2.33)	
quintile 2	1.35 (1.02–1.79)	
quintile 3	1.14 (0.84–1.56)	
quintile 4	1.05 (0.78–1.42)	
quintile 5 (ref.)	1.00	0.0005
**Sociability**		
quintile 1 (lowest)	1.77 (1.29–2.44)	
quintile 2	1.43 (1.06–1.95)	
quintile 3	1.42 (1.05–1.93)	
quintile 4	1.10 (0.78–1.55)	
quintile 5 (ref.)	1.00	0.0032
**Maternal Variables**		
**Mothers age at birth**		
Teen	1.40 (0.85–2.3)	
40+	0.67 (0.37–1.22)	
20–39 (ref.)	1.00	0.1409
**Mother alcohol problem**		
Yes	1.37 (1.07–1.75)	
No (ref.)	1.00	0.0125
**Mother smoker**		
Yes	1.67 (1.37–2.04)	
No (ref.)	1.00	<.0001
**Mother K6 symptomatic**		
Yes	1.6 (1.26–2.03)	
No (ref.)	1.00	0.0001
**Maternal Education**		
Year 12	3.87 (3.02–4.95)	
Year 11 or less	2.46 (1.93–3.13)	
University (ref.)	1.00	<.0001
**Maternal work hours**		
zero hours^b^	1.14 (0.88–1.48)	
full-time: 38 hours +	2.00 (1.64–2.44)	
part time: 1–37 hours (ref.)	1.00	<.0001
**Maternal consistency**		
quintile 1 (lowest)	2.77 (2.08–3.7)	
quintile 2	1.70 (1.26–2.29)	
quintile 3	1.27 (0.94–1.72)	
quintile 4	1.49 (1.1–2.03)	
quintile 5 (ref.)	1.00	<.0001
**Maternal inductive reasoning**		
quartile 1 (lowest reasoning)	1.2 (0.93–1.55)	
quartile 2	0.9 (0.72–1.12)	
quartile 3	0.93 (0.74–1.17)	
quartile 4 (ref.)	1.00	0.0657
**Maternal warmth**		
quintile 1 (lowest warmth)	0.96 (0.74–1.25)	
quintile 2	0.88 (0.67–1.15)	
quintile 3	0.95 (0.71–1.25)	
quintile 4	1.05 (0.82–1.35)	
quintile 5 (ref.)	1.00	0.6388
**Maternal hostility**		
quintile 1 (greatest hostility)	1.56 (1.16–2.08)	
quintile 2	1.17 (0.86–1.59)	
quintile 3	1.13 (0.81–1.59)	
quintile 4	1.08 (0.78–1.49)	
quintile 5 (ref.)	1.00	0.0113
**Family Variables**		
**Mother Non-English Speaking Background**		
Yes	2.17 (1.73–2.71)	
No (ref.)	1.00	<.0001
**Family Structure**		
Single mother family	1.56 (1.24–1.96)	
Other (ref.)	1.00	0.0002
**Number of siblings**		
One	1.06 (0.81–1.41)	
Two	1.35 (1.01–1.82)	
Three	1.8 (1.24–2.6)	
Four plus	3.97 (2.62–6.02)	
Zero (ref.)	1.00	<.0001
**Family income per week**		
Under $600	4.15 (2.92–5.91)	
$600–$999	3.61 (2.64–4.94)	
$1000–$1499	2.36 (1.7–3.28)	
$1500–$1999	2.1 (1.47–3)	
$2000 or more (ref.)	1.00	<.0001
**Health care card**		
Yes	1.88 (1.54–2.30)	
No (ref.)	1.00	<.0001
**Financial hardship**		
Yes	1.39 (1.17–1.66)	
No (ref.)	1.00	0.0002
**SEIFA disadvantage index**		
quintile 1 (lowest SEIFA)	2.97 (2.15–4.09)	
quintile 2	1.7 (1.24–2.32)	
quintile 3	1.52 (1.08–2.12)	
quintile 4	1.1 (0.77–1.57)	
quintile 5 (ref.)	1.00	<.0001
**Reads to child**		
Not at all	4.86 (3.36–7.03)	
1–2 days/ week	2.11 (1.67–2.66)	
3–5 days/ week	1.68 (1.35–2.09)	
Daily (ref.)	1.00	<.0001
**Playgroup**		
No	0.85 (0.7–1.03)	
Yes (ref.)	1.00	0.0923
**Hours a week in care**		
9–20	1.13 (0.76–1.7)	
21–30	1.28 (0.82–1.99)	
31+	0.95 (0.65–1.39)	
8 hours or less (ref.)	1.00	0.1621

a Study Child Aboriginal and/or Torres Strait Islander;

b Includes not in labour force.

Of the 29 candidate predictors, twelve were above the odds ratio cut-off of 2.0. In order of increasing magnitude of effect size these were: Low maternal work hours (2.00), NESB status (2.17), Aboriginal and/or Torres Strait Islander status (2.67), low persistence (2.75), low maternal parenting consistency (2.77), socio-economic area disadvantage (2.97), low school readiness (3.79), low maternal education (3.87), four or more siblings (3.97), low family income (4.15), child not read to at home (4.86), and low PPVT score at 4 years (5.32).

Nine candidate predictors were statistically significant in the bivariate model, but below the OR 2.0 cut-off: Problematic alcohol use (1.37), financial hardship (1.39), maternal hostility (1.56), single mother status (1.56), maternal mental health distress (1.6), maternal smoking (1.67), high temperamental reactivity (1.75), low temperamental sociability (1.77), and having a health care card (1.88).

The remaining eight candidate predictors of low PPVT at age 8 were not statistically significant in the bivariate model: male gender, low birthweight, ear infections, teenage mother status, low maternal inductive reasoning, low maternal warmth, not attending playgroup, and daily use of non-parental child care.

### Multivariate Analysis

The twelve predictors meeting the initial cut-off criterion of an odds ratio of 2.00 or higher in the unadjusted analyses were then included in the multivariate model (see Table 6).

**Table 6:** Multivariate associations between child, maternal and family characteristics and low respective vocabulary ability at 8 years.

Variables	OR	P-value
Child variables		
**PPVT group at 4 years**		
Low (bottom 15%)	3.49 (2.66–4.58)	
Middle-high (Remaining 85%) (ref.)	1.00	<.0001
**Ethnicity**		
Study Child ATSI^a^	1.23 (0.69–2.22)	
Study Child non-ATSI (ref.)	1.00	0.4832
**Who Am I?**		
quintile 1(lowest)	2.5 (1.73–3.63)	
quintile 2	2.13 (1.51–3.02)	
quintile 3	1.46 (0.96–2.21)	
quintile 4	1.63 (1.14–2.34)	
quintile 5 (ref.)	1.00	<.0001
**Persistence**		
quintile 1 (lowest)	1.65 (1.1–2.47)	
quintile 2	1.27 (0.86–1.86)	
quintile 3	1.11 (0.75–1.65)	
quintile 4	1.56 (1.05–2.33)	
quintile 5 (ref.)	1.00	0.0393
**Maternal variables**		
**Maternal Education**		
Year 12	1.61 (1.18–2.2)	
Year 11 or less	2.55 (1.81–3.58)	
University (ref.)	1.00	<.0001
**Maternal work hours**		
zero hours^b^	1.06 (0.82–1.38)	
full-time: 38 hours +	1.08 (0.76–1.53)	
part time: 1–37 hours (ref.)	1.00	0.8681
**Maternal consistency**		
quintile 1 (lowest)	1.24 (0.86–1.79)	
quintile 2	1.17 (0.79–1.73)	
quintile 3	1.2 (0.81–1.78)	
quintile 4	1.89 (1.31–2.72)	
quintile 5 (ref.)	1.00	0.0068
**Family home environment variables**		
**Mother Non-English Speaking Background**		
Yes	1.59 (1.14–2.22)	
No (ref.)	1.00	0.0067
**Siblings**		
One	1.29 (0.88–1.89)	
Two	1.58 (1.04–2.4)	
Three	1.43 (0.84–2.43)	
Four plus	2.02 (1.03–3.94)	
Zero (ref.)	1.00	0.1647
**Family income per week**		
Under $600	1.66 (1.06–2.6)	
$600–$999	1.48 (1.00–2.18)	
$1000–$1499	1.35 (0.92–1.98)	
$1500–$1999	1.5 (1.00–2.24)	
$2000 or more (ref.)	1.00	0.2059
**SEIFA disadvantage index**		
quintile 1 (lowest SEIFA)	1.78 (1.24–2.56)	
quintile 2	1.45 (0.99–2.14)	
quintile 3	1.16 (0.79–1.72)	
quintile 4	0.88 (0.58–1.32)	
quintile 5 (ref.)	1.00	0.0006
**Reads to child**		
Not at all	1.09 (0.58–2.05)	
1–2 days/ week	1.19 (0.87–1.61)	
3–5 days/ week	1.27 (0.96–1.67)	
Daily (ref.)	1.00	0.3871

a Study child Aboriginal and/or Torres Strait Islander;

b Includes not in labour force.

In the multivariate model, low PPVT at 4 years, low maternal education and low school readiness showed moderate or higher adjusted effects on the odds of low PPVT at 8 years. The odds of low PPVT at age 8 were 3.49 times higher for children who had low PPVT at age 4 relative to children who had middle-high PPVT at age 4; 2.55 times higher for children whose mothers had only completed year 11 of Australian Schooling relative to children of University educated mothers; and 2.50 times higher for children in the lowest quintile of school readiness at age 4 relative to those in the highest quintile of school readiness.

The following risk factors had adjusted effects on low PPVT at 8 years below our criterion of moderate effect size (2.0): Low maternal parenting consistency, socio-economic area disadvantage, low temperamental persistence, and NESB status. The odds of low PPVT at age 8 were 1.89 times higher for children of mothers in the lowest quintile for maternal parenting consistency relative to those children of mothers in the highest quintile of parenting consistency; 1.78 times higher for children living in the most disadvantaged neighbourhood relative to those children in the least disadvantaged neighbourhoods; 1.65 times higher for children who were in the lowest quintile of temperamental persistence relative to those in the highest quintile; and 1.59 times higher for children of mothers who spoke a language other than English relative to those children whose mother spoke English.

The following risk factors were not statistically significant in the multivariate model: Aboriginal and/or Torres Strait Islander status, low maternal work hours, low family income, child not read to at home, and one or more siblings.

#### Sensitivity-Specificity Analysis

Odds ratios and statistically significant associations do not necessarily lead to clear predictive relationships. Inevitably, researchers and practitioners turn their attentions towards asking how our knowledge of the associations demonstrated here might affect decisions about screening children for low language ability, predicting the persistence of this status, and offering interventions. Our intention in this section is not aimed at producing a practical algorithm for screening children for low language status per se. That would be premature. What we do want to do though, is widen the developmental description beyond just the empirical documentation of language growth and its associations. This entails including assessing the implications of the emerging evidence of high intra-individual variability in the growth of language, as measured here, the relatively low level of association between language measures and important predictors, and what this might portend for those interested in early detection and intervention. To do this, we include analyses of the predictive utility of these estimates.

We have provided the odds ratios for each predictor measure in Tables 5 and 6. The predictive utility of a variable in a logistic regression may be calculated not only from the odds ratio, but may also be calculated by estimating the area under the receiver operating characteristic curve, and by calculating sensitivity, specificity, positive predictive value and negative predictive value [44]. At the outset we would note that the single best predictor of low receptive vocabulary at age 8 was low receptive vocabulary at age 4 (unadjusted OR 5.32). Using just low receptive vocabulary at age 4 as a single predictor we calculated an area under the Receiver Operating Characteristic (ROC) Curve of 0.62. This may be interpreted as the probability that a randomly chosen child with low PPVT at age 8 will be correctly rated, relative to a randomly chosen child that does not have low PPVT at age 8 (that is, it is a measure of how well the model discriminates cases from non-cases) [45].

In contrast, the multivariate model estimates the probability that each study child has low PPVT at age 8, based on all of the selected candidate predictors at age 4. The multivariate model had an ROC of 0.75, and shows that the addition of other candidate predictors along with the age 4 PPVT performance improves discriminative utility – in other words, including additional predictors in the model has improved how well the model discriminates cases from non-cases. However, in order to evaluate predictive utility the researcher needs to establish a cut-point at which a child is classified as a case or non-case (that is, at what estimated probability do we predict a child will have low PPVT at age 8) [46], [47].

**Figure 2:**
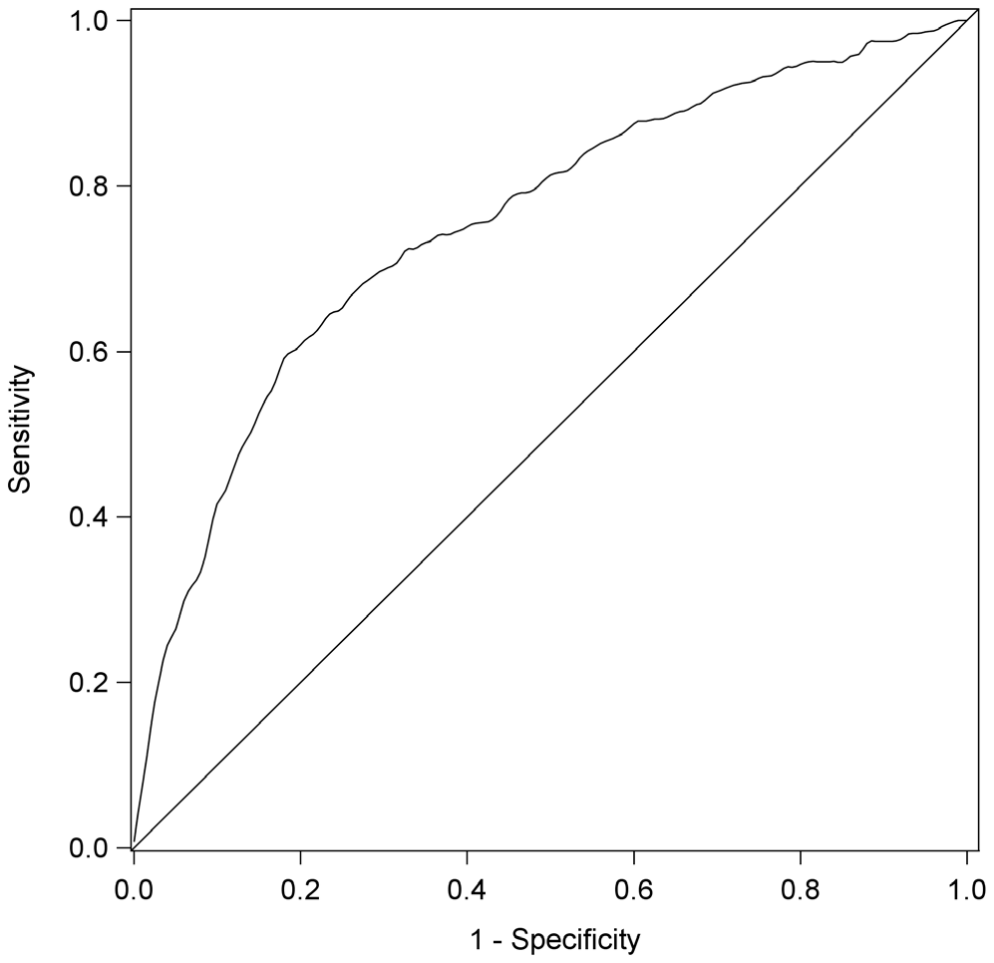
Sensitivity-Specificity Curve, Multivariate Logistic Model.

Following examination of the ROC curve (see Figure 2), a probability criterion threshold of 0.13 was chosen to give an approximately equal sensitivity (0.694) and specificity (0.711). Hosmer and Lemeshow [44] would term this ‘acceptable’ discrimination. Using this criterion, of the 402 children with low PPVT at age 8, the model correctly identified 279 (69.4%) of them and of 2,779 children without low PPVT at age 8, the model correctly identified 1,975 (71.1%) (Table 7). However determining acceptable classification discrimination is more complex than estimating the proportion of children with low PPVT at age 8 (sensitivity) and those who were not low at age 8 (specificity). The proportion of children classified as low that are actually low (positive predictive value), and the proportion of children correctly classified as not low (negative predictive value) are also of importance. The positive predictive value of the model at a probability threshold of 0.13 is much lower than the observed sensitivity and specificity; so, of 1,083 children predicted to have low PPVT at 8 years, only 279 of them actually had low PPVT (25.8%). That is, the model incorrectly predicted 804 of these children as having low PPVT at 8 years when in fact they did not. The negative predictive value is much higher, of 2,098 children predicted not have low PPVT at 8 years, 1,975 (94.1%) actually did not have low PPVT.

**Table 7:** Sensitivity-Specificity of Multivariate Logistic Model at probability cut-off of 0.13.

	Actual		
Predicted	Low PPVT at 8 years	Not Low PPVT at 8 years	Total
Low PPVT at 8 years	279	804	1083
	69.4% sensitivity (true positives)	28.9% 1-specificity (false positives)	
	25.8% positive predictive value	74.2% 1- PPV	100% positive results
			
Not Low PPVT at 8 years	123	1975	2098
	30.6% 1-sensitivity (false negatives)	71.1% specificity (true negatives)	
	5.9% 1-negative predictive value	94.1% negative predictive value	100% negative results
**Total**	**402**	**2779**	**3181**
	**100% cases**	**100% non-cases**	

So, at a practical level, if decisions were being made about offering ongoing professional monitoring of developmental status, or offering intervention, our model is good at predicting those children who will not have low receptive vocabulary status at age 8. In other words, we know who is less likely to need monitoring or intervention. Admittedly, of the children we predicted at age 4 to not have low receptive vocabulary at age 8, we will have misclassified 5.9% of these who actually go on to have low receptive vocabulary at age 8. This might be deemed a tolerable miss-rate. In contrast though, for the children at age 4 who were predicted to go on to have low receptive vocabulary at age 8, 74.2% of these children would actually go on to be classified as not having low receptive vocabulary at age 8. In other words, it is possible that considerable resources will be deployed towards children who, in all likelihood, won't need intervention. Decisions would need to be made as to whether this represents a tolerable deployment of monitoring and/or intervention to assist the 25.8% of children classified at age 4 who would otherwise have a low receptive vocabulary status at age 8.

We would note that it is possible to select different threshold criteria for classification. Changes in the selection of the threshold criterion produce changes in onward sensitivity and specificity, and these in turn, affect positive and negative predictive values. Relaxing the threshold identifies at age 4 more of the children who actually go on to be classified as having low receptive vocabulary at age 8, but at the consequence of identifying a larger number of ultimately unaffected children. For example, a cut-off of 0.05, increases the sensitivity (92.2%), decreases specificity (28.2%), decreases positive predictive value (15.7%), and increases negative predictive value (94.3%). In contrast, increasing the threshold criterion to 0.20 decreases the sensitivity (50.0%), increases specificity (86.1%), increases positive predictive value (34.1%), and decreases negative predictive value (92.2%). While our criterion was selected to balance sensitivity and specificity, the establishment of the threshold criterion rests with decisions and values about the cost and effectiveness of monitoring and/or treatment, the outcome benefits, and the consequences of not intervening.

## Discussion

Vocabulary acquisition is a major component of language development and a central marker of the semantic system. In this study we describe the extent to which low language ability, as measured by receptive vocabulary, persists or improves beyond toddlerhood, and the extent to which other factors in the child's wider developmental environment are associated with this in the preschool and early school years. To do this, we estimated the extent to which low receptive vocabulary at 4 years was associated with onward low receptive vocabulary at 8 years and estimated the contribution of child, maternal and family risk factors to predicting from age 4 onward low receptive vocabulary at 8 years.

The results show substantial variability in receptive vocabulary performance in the epoch from age 4 to age 8 with weak – at best, modest – contributions from child, maternal and family factors. This finding in children aged 4 to 8 mirrors the existing studies demonstrating high variability in vocabulary acquisition in children in the toddler range. The poor positive predictive value of persistent low language ability from toddlerhood has been attributed to the immaturity of the emergent language system whereby low language ability is a transient variant of typical language development for most children. The results of our study suggest that, for most children, low receptive language ability at 4 years is also a transient variant of typical language development. Therefore, the catch-up pattern observed in follow-up studies of two-year-olds, was also evident in this study of language outcomes for four-year-olds.

Our findings also demonstrate that the configuration of risks for low receptive vocabulary ability from 4–8 years was quite different to the configuration of risks in studies of toddlers. In toddler studies, maternal and family environment risk factors are conspicuous for their absence. Instead, child risk factors dominate the etiological models. These risk factors are subtle and best characterised as nonclinical neurobiological vulnerabilities that result in a slower start in language acquisition [48], [49]. In this study, parental and family level risks were predominant, suggesting that these factors are increasingly important over time [50].

There are limitations to these findings.

First, in developing our multivariate logistic model, we set an odds ratio of 2 to represent a criterion effect size of substantive interest. This is not a commonly used device, particularly with logistic regression, and other approaches in predictor selection and retention could have been taken. For example, researchers commonly choose to include all statistically significant variables initially screened for subsequent entry to the multivariate model. To test the robustness of our approach, we repeated the analysis and included all statistically significant variables from the bivariate modelling into a multivariate model, and then used a stepwise elimination of non-significant variables until a final model was achieved. The results of this analysis were consistent with the results presented in this paper. There were no substantial changes in the pattern of effects or in the model's predictive utility. To test whether the model's predictive utility could be improved by a more inclusive set of predictors, and by treating predictors as continuous rather than categorical where appropriate we also developed a predictive logistic model using all 29 candidate predictors. This model represented a marginal improvement on the final model presented in this paper, but did not change the pattern of findings in a meaningful fashion.

Second, our modelling only permits examination of the predictive utility of a single language component – semantics, as measured by receptive vocabulary. We accept that a clinical diagnosis of language impairment is based on psychometric assessment of multiple dimensions of language as well as naturalistic observation and information about the child's communication in the home and other environments such as school. We do not recommend our model as a clinical screening tool, but as an illustration of the difficulties in effectively implementing screening in a population representative sample based on a broad range of child, maternal and family characteristics, without extensive and time-consuming testing. Further, different dimensions of language (e.g., grammar vs. semantics) have different growth characteristics that produce different patterns of growth over time. For example, children's acquisition of grammar proceeds in known steps towards the adult grammar. This means that children's performance can be interpreted in relation to progress towards a known developmental endpoint – that is, mastery of the adult grammar [51]. By contrast, receptive vocabulary acquisition does not have a developmental endpoint and can only be referenced to the normative performance of children the same age, as in this study. This raises the question of whether the poor prediction of low language ability and the catch-up pattern observed in this study was specific to only the dimension of language we assessed, that is, receptive vocabulary. We think this is unlikely. Findings from a study that comprehensively measured both the semantic and grammar dimensions of language revealed that most children with a history of low language ability at 2 years, met normative expectations on measures of grammar and semantics at 7 years [52]. Notwithstanding this, group differentiation was greater on measures of grammar than receptive vocabulary.

Third, it could be argued that interests in and concerns about the persistence of low language ability and its prediction in the period from 4 to 8 focusses “too late” in the language development trajectory. And yet, what we demonstrate here is that the high variability in language acquisition and the weak predictive associations about language development empirically documented in infants and toddlers is also being observed in the epoch from 4 years to 8 years. This is substantively important in shaping our thinking about the nature of language variability. It is also the case that what happens in language development from 4 to 8 years is equally important in terms of onward lifecourse outcomes and has a direct bearing on readiness to learn.

Fourth, it could be argued the predictive utility of our models would have been enhanced had we included more extensive measures of child cognitive or language ability at age 4, or if we had combined measures into composites to reduce measurement error. We cannot comment in detail on the predictive utility of measures which were unavailable to us, but would note that the variables included in the LSAC were based upon known risk factors from established longitudinal studies and a consideration of those variables which could meaningfully impact policy interventions [25]. It would not be appropriate to collapse these measures into composites. Further, we would note that although composites can potentially reduce measurement error, the disadvantages of composites include difficulty in differentiating the independent and interactive effects of each component the loss of potentially valuable information [53].

Finally, it could be argued that as we have no measure of any remedial treatment, speech therapy or other interventions which children in our study have received between ages 4 and 8 we understate the efforts of parents and educators in assisting those children who started with low receptive vocabulary. If our model had included measures of intervention between ages 4 and 8, we would likely have a stronger predictive model of receptive vocabulary at age 8. However, whilst this seems a plausible limitation, this study was explicitly aimed at examining predictors from age 4 on age 8 receptive vocabulary. Further, this does not change our thesis that variability in children's language development is an important developmental phenomenon, and that the predictive utility of models of language is under-examined.

Notwithstanding these limitations, what are the implications of these findings?

First, much of the extant literature on language development trajectories is focussed on documenting the typical variability in language growth and assessing its predictors. This work is relatively silent about the utility of these predictors. Some of this reticence reflects methodological constraints which preclude offering these insights. But where there are good longitudinal data, of well described samples and populations, with relatively robust and replicated measures, some advance should be made in extending the implications of the findings towards demonstrating what they might practically mean. How do these associations guide planning, funding, and arranging services and interventions?

In prompting for more of this, we would suggest that the high developmental variability in receptive vocabulary performance from age 4 to age 8 will make accurate identification of children at age 4 for intervention on the basis of their low receptive vocabulary performance and a mix of child, maternal and family risks, challenging. We found the strongest predictor of low receptive vocabulary at age 8 was low receptive vocabulary at age 4. However, despite a moderate odds ratio with vocabulary ability at 8, receptive vocabulary was nonetheless a limited predictor of persistently low receptive vocabulary ability at 8 years. Dale et al., reported comparable findings, in a population level sample of young twins from 2–4 years [54] and 4–12 years [55]. Their conclusion from these studies, consistent with ours, was that prediction of persistent language impairment is not accurate enough to justify screening to identify children who are likely to have future language impairment. This means that identifying preschool age children for targeted vocabulary intervention will mostly include children who move within the typical range of language ability in the early years of school. On the other hand, not all children will catch up and this group of children will likely require ongoing support for language learning through the school years.

In addition to low age 4 receptive vocabulary, we found that a well-fitted multivariate model featuring risks of substantive magnitude does not do particularly well in improving prediction of low onward receptive vocabulary ability at age 8. While we are not proposing that the specific model we have fitted here be used for screening general populations, the empirical literature on the prediction of language outcomes for unselected populations of children is not encouraging [56]. Is it likely that we will see models developed with greater predictive utility? What if this is as good as it gets? These are unanswered questions for future studies. Future studies could investigate the classification accuracy of predicting language status at a later age (e.g., age 8) from two or more measures of low language performance at younger ages (e.g., age 4 and age 6), as opposed to a single measure of low language performance at an earlier age (e.g., age 4).

Finally, some readers may be concerned that we are dismissive of the risks for persistent language difficulties. This is far from the case. In a recent Australian study, the financial costs associated with parents accessing health services for pre-schoolers with communication disorders were substantial [57]. In two related studies, substantial over- and under-servicing were identified [58], [59]. In an RCT of a population-based early language promotion program, language outcomes for children in the control arm met normative expectations on psychometric tests of expressive and receptive language [60]. These are confronting findings and speak to the need for well-judged approaches to responding to need.

The emergent finding in studies of language growth trajectories is that variability in language growth needs to be seriously considered. Those who start below age expectations may not be those who continue to stay behind age expectations. Children need to be monitored throughout their development. The “best” predictor may actually be the persistence of low language performance over time rather than status at a given point in time. Our findings illustrate the tension between stability and change over time. This needs to be considered when observing developmental phenomena, particularly in a population representative sample rather than a sample of children already identified as ‘at risk’. Whilst it is difficult to predict with great accuracy which children will have poor language at age 8, it is also clear that some children are exposed to far greater risk than others.

### Future research

By school age, English-speaking children have reached adult levels of competence in some dimensions of language (e.g., the speech sound system, clause structure) while other dimensions such as receptive vocabulary ability continue to develop throughout life. Once children start formal schooling, learning to read and write become new dimensions of the language system. While oral language is the substrate for literacy, literacy also facilitates receptive vocabulary knowledge by giving children access to experiences, and words to describe those experiences, beyond the day to day. It will be important for future research to determine the extent to which low receptive vocabulary ability from 4 to 8 years projects to low literacy ability and the configuration of risks for low literacy ability.
